# Data Fusion Using Improved Support Degree Function in Aquaculture Wireless Sensor Networks

**DOI:** 10.3390/s18113851

**Published:** 2018-11-09

**Authors:** Pei Shi, Guanghui Li, Yongming Yuan, Liang Kuang

**Affiliations:** 1 School of IoT Engineering, Jiangnan University, Wuxi 214122, China; njxk_sp@sina.cn; 2 Freshwater Fisheries Research Center, Chinese Academy of Fishery Sciences, Wuxi 214081, China; Yuan@ffrc.cn; 3 School of IoT Engineering, Jiangsu Vocational College of Information Technology, Wuxi 214153, China; kuangliang.89@163.com

**Keywords:** wireless sensor networks, data fusion, support degree function, dynamic time warping, sensor-cloud, water quality monitoring

## Abstract

For monitoring the aquaculture parameters in pond with wireless sensor networks (WSN), high accuracy of fault detection and high precision of error correction are essential. However, collecting accurate data from WSN to server or cloud is a bottleneck because of the data faults of WSN, especially in aquaculture applications, limits their further development. When the data fault occurs, data fusion mechanism can help to obtain corrected data to replace abnormal one. In this paper, we propose a data fusion method using a novel function that is Dynamic Time Warping time series strategy improved support degree (DTWS-ISD) for enhancing data quality, which employs a Dynamic Time Warping (DTW) time series segmentation strategy to the improved support degree (ISD) function. We use the DTW distance to replace Euclidean distance, which can explore the continuity and fuzziness of data streams, and the time series segmentation strategy is adopted to reduce the computation dimension of DTW algorithm. Unlike Gauss support function, ISD function obtains mutual support degree of sensors without the exponent calculation. Several experiments were finished to evaluate the accuracy and efficiency of DTWS-ISD with different performance metrics. The experimental results demonstrated that DTWS-ISD achieved better fusion precision than three existing functions in a real-world WSN water quality monitoring application.

## 1. Introduction

Wireless sensor networks (WSN) have the advantage of flexible deployment, wide distribution, low cost, small volume, and have been widely applied in various fields, such as military applications (e.g., military surveillance), and civil applications (e.g., industrial surveillance, agriculture monitoring and medical monitoring) [1,2,3,4]. In aquaculture, the sensor nodes can collect water quality parameters of temperature, humidity, and dissolved oxygen constantly in the monitoring area. However, the difference between these monitoring data exists due to uneven distribution, especially in a large monitoring area. The monitoring data in a single location cannot represent the real situation of the whole monitoring area. Parallel monitoring of multiple sensors measurement is necessary [5]. The limitations of battery volume, computation ability, and communication bandwidth can influence the performance of WSN [6]. The data stream in WSN has the features of large amount, large variety, high production rate, authenticity and value. Data loss and data exceptions often occur because of the sensor or link fault, or environmental events [7]. Sensor-cloud can overcome the weaknesses of limited storage capacity, limited handling capacity and energy. Meanwhile, nodes fault repairing and error data detecting also can be solved with low cost [8]. Thus, processing large amounts of sensor data in WSN cloud system is an urgent issue [9]. It also becomes essential to introduce a handling mechanism into WSN monitoring system based on cloud platform [10]. Once abnormal data is detected, a monitoring system needs to handle it. In this study, we use data fusion mechanism to generate correct data to replace the abnormal data.

Data fusion is one of data processing techniques to reduce the data redundancy and improve the data quality [11,12,13,14,15,16,17,18,19,20]. Data fusion can be based on different theories, such as the artificial neural network fusion algorithm (ANN), fuzzy set theory, rough set theory, Dempster-Shafer evidence theory (DS), Bayesian fusion algorithm, Kalman filter theory, weighted average fusion algorithm, etc. Although most of these algorithms are able to eliminate the data redundancy and improve data quality, limitations still existed when applied in practical cases. For instance, the assumption always exists that the sensor nodes or sink nodes are functioning properly, and generating accurate data [21]. This assumption is unrealistic, since a complex environment may cause the sensor fault or data error.

This paper presents a data fusion method based on DTW time series segmentation strategy DTWS-improved support degree (ISD) function to accurately and efficiently fuse monitoring data for enhancing data quality. In weighted fusion method, DTWS-ISD combines the ISD function and Dynamic Time Warping (DTW) time series segmentation strategy together. ISD function can reduce the complexity of support degree function, thus, it is adopted to obtain the mutual support degree of sensors. DTW distance can explore the continuity of monitoring data and avoid missing some key information, and time series segmentation strategy can reduce data dimension and time consumption. DTW time series segmentation strategy is utilized to calculate the similarity between time series and replace the Euclidean distance. One advantage of DTWS-ISD is that it can enhance the network performance of WSN, reduce the data redundancy, and improve sensor data quality. Another advantage of DTWS-ISD is that it can improve the multiple sensors fusion precision and improve the fusion efficiency. The experimental results demonstrate that DTWS-ISD achieves higher precision and efficiency than three existing functions (Gauss function [22], D function [23] and SN function [24]) in the real-world aquaculture application.

The remainder of this paper is organized as follows: Section 2 presents the related work. Section 3 describes the data fusion mechanism for enhancing data quality. In Section 4, we conduct several experiments to verify weighted fusion with DTWS-ISD and present the experiment results. Finally, Section 5 gives the conclusions.

## 2. Related Work

The traditional WSN just collects data from sensors located in some specialized regions, and does not analyze the data stream. However, when used in earthquakes, forest fire prevention and water quality control, etc., we need early warming to make coping strategy. Thus, a high quality data stream and high handling capacity are also required in these applications. The application of sensor-clouds utilized the cloud computing to complete the analysis, and processing of data quickly, on a high-performance cluster [25]. Information, such as water quality monitoring, data streams of water temperature and dissolved oxygen, are collected by WSN, and sent to cloud platform for further analysis. The user can master the water quality status without leaving home.

There are many fusion technologies that can be used on a cloud platform in order to obtain high quality data. Existing data fusion technologies can be classified into two types: One consists of weighted average fusion algorithm, Bayesian fusion algorithm, Kalman filter and Dempster-Shafer evidence theory, etc. The other type includes an artificial neural network fusion algorithm, rough set theory and fuzzy set theory, etc.

Artificial neural network (ANN) can analyze the nonlinear system problem well, but the complex structure and random parameter can lead to the instability of an algorithm. The performance of Elman ANN with different configurations was discussed when handling the multi-sensor data fusion in Reference [11], and the importance of parameter selection is also proved. Fuzzy set theory is suitable for processing the sensor data which is incomplete or in an uncertain state by self-learning continuously. A multi-sensors data fusion technique was developed by using fuzzy clustering that is based on the ability of fuzzy sets in dealing with imprecision and uncertainty [12]. Rough set theory is also suitable for dealing with uncertain or unclear data, but has been limited to the attribute reduction for many years. Different applications of rough set theory in information fusion were presented in Reference [13]. DS theory has an advantage of studying nondeterministic problems in data fusion. But when dealing the conflict data, an abnormal value will occur frequently. A fusion-based uncertainty aware sensor networks deployment problem was discussed in Reference [14], DS theory was used to define a generic evidence fusion scheme that captures several characteristics of real-world applications. Before fusing the multi-sensors data, Bayesian network algorithm requires prior knowledge, and obtains the prior probability distribution to calculate the reliabilities of sensors. A novel approach for fault detection taking advantage of the mathematical framework of Bayesian to integrate micro and macro data was presented in Reference [15]. A Kalman filter can deal with the redundant information, but the prior knowledge and model of the target is required. A novel multi-sensor optimal data fusion methodology based on adaptive fading unscented Kalman filter for multi-sensor nonlinear stochastic systems was proposed in Reference [16]. As for the weighted fusion algorithm, it realized the weighting operation of data streams after calculating weights of sensors. A novel two classes of the ordered weighted gradient fusion algorithm was discussed in Reference [18] to fuse the multi-scale information inspired by the human visual system.

Weighted fusion algorithm is used in many application fields. It does not require the prior knowledge of sensor system during fusion, and can realize high precision information fusion with sensor data. Yager [22] proposed a power mean average operator to fuse sensor data based on the calculation of support degree. This algorithm can be applied in real-time fusing for its high efficiency. Xiong [23] provided a new model support function in real-time data fusion based on grey correlative degree theory. Before operating data fusion, it required checking for data consistency and exponentially smoothed three times on sensor nodes to improve data quality. Besides, Duan [24] adopted the regression prediction method based on siding window to check the consistency of data and provided a homogeneous data weighted fusion algorithm based on improved support degree to fuse these homogeneous data.

Although there were other works [20,26] that used the weighted fusion algorithm, based on different support degree functions, weighted fusion is preferred, due to the easy computation. The computational complexity and precision of these support degree functions are critical issues to be solved. In this paper, weighted fusion algorithm based on DTWS-ISD function was proposed to enhance data quality in aquaculture WSN. The first step of enhancing data quality is to collect data from a monitoring system. Then, finish checking data consistency to eliminate error. Finally, adopt data fusion mechanism to generate fused data.

## 3. Enhancing Data Quality Based on Data Fusion Mechanism

### 3.1. Overview of Data Correction

Fault detection and data fusion mechanism are the crucial steps for improving the dissolved oxygen data quality. Due to the high correlation of multi-sensors data in time and space, it is necessary to check the consistency of historical data firstly. On the basis of reconstructing missing data and detecting the outlier, a new data set can be obtained to finish data fusion.

**Figure 1:**
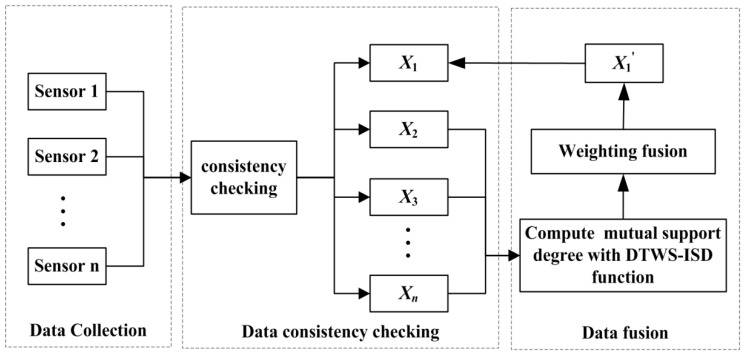
The flow chart of data correction.

When any sensor does not work, data fusion mechanism can help to obtain corrected data to replace abnormal data. Suppose Sensor 1 is the fault node, the first step is to compute the mutual support degree values of sensors with DTWS-ISD function. Then, compute the fused result based on weighted fusion method. Each sensory data need to execute the following data processing mechanism to improve data quality as shown in Figure 1. The DTW method and time series segmentation strategy are adopted together to improve ISD function during the mutual support degree computing process.

From Figure 1, when Sensor 1 is the fault node, we can get a new dataset *X_i_* = [*x_i_*_1_, *x_i_*_2_, *…*, *x_it_*] in consistency checking module. Fusion module will utilize the fusion result of sensor nodes *X*_2_ = {*x*_21_, *x*_22_, …, *x*_2*t*_}, *X*_3_ = {*x*_31_, *x*_32_, …, *x*_3*t*_} and *X_n_* = {*x_n_*_1_, *x_n_*_2_, …, *x_nt_*} to correct *X*_1_ = {*x*_11_, *x*_12_, …, *x*_1*t*_}. Here, *x_ik_* represents the observed value of Sensor *i* in time *j* after data consistence checking, *i* = 2, 3, …, *n*, and *k* = 1, 2, …, *t*.

Algorithm 1 explains the detailed process of data correction. The input of data correction algorithm is the original data of dissolved oxygen content *o_i_*, which is collected from *n* sensors, as well as the time length *t* used for time series segmentation. The output of the algorithm is *X_Fuse_*, which is applied to correct the data stream and improve data quality. The inner loop (lines 3–7) obtains the support degree matrix *s_ij_* to compute the weights of sensors *w_j_*. In fact, the support degree value is limited to the *Dist* value between Sensor *i* and Sensor *j*. All these calculations are influenced by the *X_i_*, which is obtained from data consistency checking. Thus, in the inner loop, each subsequence only needs to be performed *j* − 1 times when Sensor 1 is the fault node. We got used result *X_Fuse_* with the calculation in lines 8-9 to replace the error data *X*_1_.


**Algorithm 1. Data Correction**
**INPUT:** Original data of dissolved oxygen content, O = {*o*_1_, *o*_2_, *…*, *o_n_*};**OUTPUT:** Fused Data (*X_Fuse_*);1:
**BEGIN**
2:  *X_i_* = [*x_i_*_1_, *x_i_*_2_, *…*, *x_it_*]←consistency checking of *o_i_*;3:  **for**
*i* = 1, *j* = 2:*n*4:  compute *Dist*(*X_i_*, *X_j_*);5:  *s_ij_* = DTWS-ISD(*X_i_*, *X_j_*);6:  *w_j_* = *s_ij_*/*sum*(*s_ij_*);7:  **end for**8:  $X_{1}^{\prime }(T)=\sum_{1}^{j}\omega_{j}\cdot X_{j}$;9:  *X_Fuse_* = *X*_1_′;10:
**END**
11:**Return***X_Fuse_*;

### 3.2. Data Consistency Detection

Due to the instability and transmission errors of underwater sensors in aquaculture, data exception or data missing often happens. Fragmentary data is mended from dissolved oxygen sensors by linear interpolation method [27]. Equation (1) shows the calculation process.

(1) $$o_{k+i}=o_{k}+\frac{i\times (o_{k+j}-o_{k})}{j},0<i<j,i,j=1,2,\ldots ,n,$$

where *o_k_* is the observed value of dissolved oxygen content in time *k*, *o_k_*_+*j*_ is the observed value of dissolved oxygen content in time *k + j*, *o_k_*_+*i*_ is the missing value in time *k + i*.

Consistency detection is realized by Autoregressive Integrated Moving Average Model (ARIMA) [28]. The main steps of consistency detection are described as follows:

Step 1: Analyze the correlation of dissolved oxygen time series data and test the data stability.

Step 2: Determine the auto regression order *p* and moving-average order *q* of ARIMA. Build the optimal ARIMA model on the basis of these parameters.

Step 3: Calculate the prediction interval (PI) to determine whether the data collected is abnormal.

*o_i_*(*t*) = *x_i_*(*t*) + *C.* (2)

Here, *o_i_*(*t*) is the multi-sensor data in time *t*. *x_i_*(*t*) is the estimated value of ARIMA. *C* is the cost function [29]. Equation (3) calculates the PI value on the basis of the estimated value *x* of ARIMA.

(3) $$PI=x\pm t_{\alpha /2,n-1}\times s\times \sqrt{1+1/n}$$

where *t* is the *P*% of a Student’s *t*-distribution with *n* − 1 degree of freedom, *n* is the sample size, and *s* is the standard deviation of *n* samples.

### 3.3. The Support Function

Weighted fusion method is one of the popular algorithms to fuse the homogeneous data [30]. Support function is used to explore the correlation between sensors from the experimental dataset. To express the support function well, we describe some useful parameterized formulations. Let sup(*a*, *b*) be the proximity between two elements *a* and *b*, called support degree. It meets the following properties:

(1) sup(*a*, *b*) ∈ [0, 1]
(2) sup(*a*, *b*) = sup(*b*, *a*)
(3) If |*a* − *b*| < |*x* − *y*|, then sup(*a*, *b*) > sup(*x*, *y*),  *a*, *b*, *x*, *y* > 0

Actually, the more similar or closer the two elements, the more they support each other. Based on the three properties, Yager proposed the binary support functions [22], which is a discontinuity. One common form of the support function with a continuous is the Gaussian support function, which is defined as:

(4) $$\begin{array}{l} \sup(a,b)=G(a,b,K,\beta )=K\times e^{-\beta \cdot {\left(a-b\right)}^{2}}\\ K\in [0,1],\beta \geq 0 \end{array}$$

where *K* is the maximally allowable support and can control the amplitude of the function. *β* is acting as the attenuation factor of function. The larger the *β* the more meaningful differences in distance. It should be noted that *a = b* makes the sup(*a*, *b*) *= K.* Thus, the distance between *a* and *b* will get larger, sup(*a*, *b*)→0. Gaussian support function is symmetric and lies in the unit interval. The calculation of this sup(*a*, *b*) relies on the exponent operation.

### 3.4. Weighted Fusion Based on Improved Support Degree

#### 3.4.1. Improved Support Degree

To reduce the high computational complexity in calculating the Gaussian support degree function, a novel ISD function based on the theory of grey incidence analysis [31] is proposed in this paper. Liu [32] utilized the theory of grey incidence analysis to represent the proximity of two elements. Inspired by this idea, we constructed the ISD function by replacing the exponent operation of Gaussian support function. The computational complexity of support function can be reduced. The ISD function is defined as:

(5) $$\begin{array}{l} \sup(a,b)=ISD(a,b,K,\beta )=K\times {\left(1+\beta |a-b{|}^{2}\right)}^{-1}\\ K\in [0,1],\beta \geq 0 \end{array},$$

where *K* decides the amplitude of the function, *β* denotes the attenuation factor of the function. If *K* is fixed, the attenuation velocity of support degree will go up with *β*. The smaller the difference between two elements is, the higher the support degree value is.

**Figure 2:**
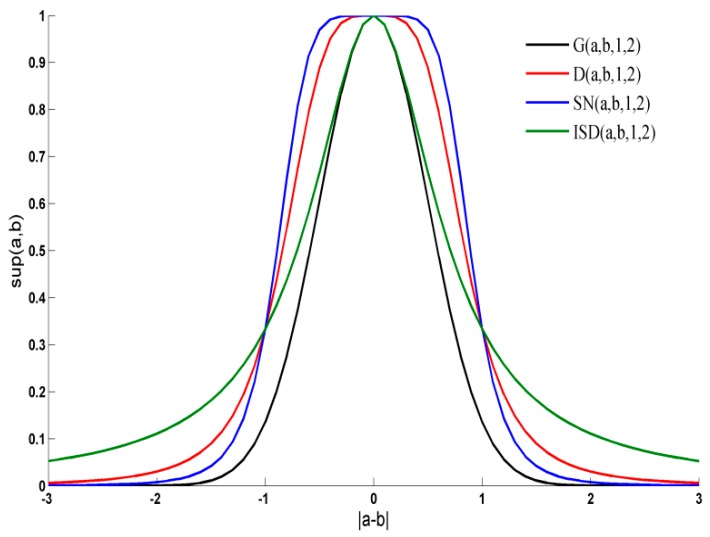
The comparison of different support degree functions.

Usually, the difference of dissolved oxygen content data at the same depth in an aquaculture concrete tank is lower than 2 mg/L, that is |*a* − *b*| ∈ [−2, 2]. To know the difference between support degree functions, a characteristic curve comparison of these functions is done in Figure 2. The G(*a*, *b*, 1, 2), D(*a*, *b*, 1, 2), SN(*a*, *b*, 1, 2) and ISD(*a*, *b*, 1, 2) represent the characteristic curves of these functions when *K* = 1 and *β* = 2.

Figure 2 showed that ISD(*a*, *b*, 1, 2) function can approximate the Gaussian support function G(*a*, *b*, 1, 2) well. The approximation effect of ISD support degree is much better than other support degree functions when |*a* − *b*| ∈ [−1, 1]. Actually, most of dissolved oxygen difference values are in [−1, 1]. For simplicity, let parameter *K* = 1, *β* = 2.

#### 3.4.2. Improved Support Degree Function Based on DTW Distance (DTW-ISD)

The traditional support function is widely used to measure the proximity between two elements at time *t*. However, the Euclidean distance *d_ij_* = |*x_i_* − *x_j_*| of two elements at *t* always loses the connection information of time series data. Considering the continuity of the time series, the similarity of time series was introduced to the ISD function.

DTW distance is a prevalent algorithm for measuring the similarity between two time series which may vary in time or speed. Based on the advantages of robust to the time warping and phase–shift, DTW was introduced to the ISD function [33,34]. Thus, we obtain the DTW-ISD support function, which is defined as:

(6) $$\begin{array}{l} \sup(X,Y)=\text{DTWS-ISD}(X,Y,K,\beta )\\ =K\times {\left(1+\beta \times Dist{\left(X,Y\right)}^{2}\right)}^{-1}\\ K\in [0,1],\beta \geq 0 \end{array}$$

where *Dist* denotes the DTW distance between two time series *X* and *Y*. The time series *X* = {*X*_1_, *X*_2_, …, *X_p_*, … *X_m_*} of length *m*, and *Y* = {*Y*_1_, *Y*_2_, …, *Y_q_*, … *Y_n_*} of length *n*. Before calculating the DTW distance, the distance matrix *D_m_*_×*n*_ is constructed firstly, where the element of the matrix, (*p*, *q*), corresponds to a distance function of the squared distance between *X_p_* and *Y_q_*:*d_pq_* = (*X_p_* − *Y_q_*)^2^ [34]. A warping path maps the elements of *X* and *Y* through matrix with minimal cumulative distance between them. Then the DTW distance *Dist* is calculated as Equation (7), which corresponds to the path with minimal warping cost.

(7) $$S(X,Y)=Dist=\min\left\{\frac{1}{k}\sqrt{\sum_{k=1}^{K}w_{k}}\right\}$$

where *w* = {*w*_1_, *w*_2_, ..., *w_k_*} denotes the warping path, *w_k_* = (*p*, *q*)*_k_* denotes the *k*-th element of *w*. The warping path satisfies these constraints including boundary condition, continuity condition and monotonicity [35]:

(1) Boundary condition: The warping path from *w*_1_ = (1, 1) to *w_k_* = (*m*, *n*).
(2) Continuity condition: The steps are confined to the points in the distance matrix with *a* − *a*′ ≤ 1 and *b* − *b*′ ≤ 1, *w_k_ =* (*a*, *b*) and *w_k_*_−1_ *=* (*a*′, *b*′)
(3) Monotonicity condition: For *w_k_ =* (*a*, *b*) and *w_k_*_−1_ *=* (*a*′, *b*′), *a* − *a*′ ≥ 0 and *b* − *b*′ ≥ 0.

Therefore, the warping path can be determined using dynamic programming, as the following recurrence:

(8) $$Dist(X,Y)=d_{pq}+\min\left(Dist(X_{p-1},Y_{q}),Dist(X_{p},Y_{q-1}),Dist(X_{p-1},Y_{q-1})\right),$$

where *Dist* (*X*, *Y*) is the sum of current *d_pq_* and the minimum cumulative distance from previous elements, *d_pq_* is the current cell distance.

#### 3.4.3. ISD Function Based on DTW Distance and Time Series Segmentation

DTW has very high measure precision, but high computational complexity limits its application. DTW-ISD support function is a time-consuming process for the high dimensional calculation [36]. Therefore, we divide the time series into several subsequences by time series segmentation to reduce the complexity of the algorithm, and increase the efficiency.

We set a fixed length of segmentation for time series, and segmented them into some same-length subsequences. The DTW distance algorithm will be applied on each subsequence [37,38]. If the length of segmentation is *l*, the time series *X* and *Y* will be divided into the *m*/*l* and *n*/*l* subsequences respectively. Then calculate the DTW distance of these sequences and get the variable as follows.

(9) $$Dist\left(T\right)=Dist_{T=l}(X,Y)$$

where *Dist_T=l_*(*X*, *Y*) is the *Dist*(*X*, *Y*) in time slot *T*. *Dist*(*T*) represents the similarity distance between the series *X* and *Y*.

A combination of DTW distance and segmentation strategy (DTWS) is proposed to optimize the ISD function. DTWS takes the subsequence to calculate similarity distance and obtain the mutual support degree. Rather than on the original time series, it is computed on the segmented time series. The computational complexity of DTW is O(*mn*), and the computational complexity of DTWS is O(*l*^2^) when *m* equals to *n*. If *m* ≠ *n*, the computational complexity of DTWS is O(*|m* − *n*|%*l* × *l*).

Let *X_i_*(*T*) and *X_j_*(*T*) be the collected data from sensors *i* and *j* in time interval *T* after data consistency checking. Then substitute these data into Equation (6), we can get the DTWS-ISD support function as follows:

(10) $$\begin{array}{l} \sup(X_{i}(T),X_{j}(T))=\text{DTWS-ISD}(X_{i}(T),X_{j}(T),K,\beta )\\ =K\times {\left(1+\beta \times Dist{\left(X_{i}(T),X_{j}(T)\right)}^{2}\right)}^{-1} \end{array}.$$

### 3.5. Data Fusion Based on DTWS-ISD Function

We use the form of Equation (10) to define the proposed support function. The mutual support degree *s_ij_* between time series within time interval *T* can be constructed as follows:

(11) $$s_{ij}=\sup(X_{i}(T),X_{j}(T))=\text{DTWS-ISD}(X_{i}(T),X_{j}(T),K,\beta ).$$

Then the mutual support degree matrix can be written as follows:

(12) $$s_{ij}=\left[\begin{array}{cccccc} s_{11} & s_{12} & \ldots & s_{1j} & \ldots & s_{1h}\\ s_{21} & s_{22} & \ldots & s_{2j} & \ldots & s_{2h}\\ \ldots & \ldots & \ldots & \ldots & \ldots & \ldots \\ s_{i1} & s_{i2} & \ldots & s_{ij} & \ldots & s_{ih}\\ \ldots & \ldots & \ldots & \ldots & \ldots & \ldots \\ s_{h1} & s_{h2} & \ldots & s_{hj} & \ldots & s_{hh} \end{array}\right],$$

where *h* denotes the number of sensors. The total support degree of the other *h* − 1 sensors to Sensor *i* within time interval *T* can be expressed as:

(13) $$sum\;\text{DTWS-ISD}\left(X_{i}(T)\right)=\sum_{i\neq j}^{h}s_{ij}.$$

Let *w_j_* represent the weighted factor of Sensor *j*.

(14) $$\omega_{j}=s_{ij}/\sum_{i\neq j}^{h}s_{ij},$$

Combined with the weighted fusion strategy, the final fusion estimation value is given in Equation (15).

(15) $$X_{i}^{\prime }(T)=\sum_{j\neq i}^{n}\omega_{j}\times X_{j},$$

## 4. Experiments

### 4.1. Data Preparation

#### 4.1.1. Data Collection

**Figure 3:**
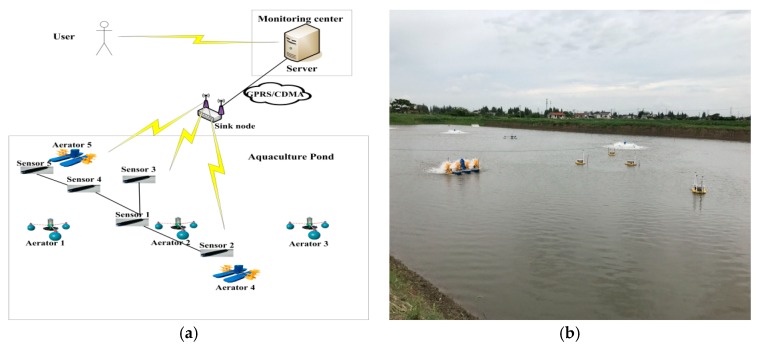
The topology of wireless sensor networks (WSN) monitoring system (**a**) and the field deployment of sensors (**b**).

All the dissolved oxygen data are collected by a WSN monitoring system monitoring system. The monitored pond is located in Changshu city, Jiangsu province. The total area of the Changshu aquaculture pond was 1.63 acres (about 110 × 60 m^2^). There are five dissolved oxygen sensors distributed in different locations of the aquaculture concrete tank. All sensors are deployed in depth of 0.5 m underwater. The collected data set includes 720 data points (sampled 10 min once) of a cleaning period from 24 May to 28 May 2017. The detailed deployment diagram is shown in Figure 3.

As shown in Figure 3, there are five sensor nodes and five aerators deployed in different locations. Aerator 5 and aerator 4 are controlled by the monitoring data of Sensor 5 and Sensor 2 respectively. Sensor 1 controls Aerator 1, Aerator 2 and Aerator 3. The data collected by sensor nodes are transmitted to sink node by wireless mode. Sink node can fuse all the sensor data and send them to server. Since all the data are stored on server, user makes control decisions through accessing the server. When any sensor does not work, data fusion mechanism can help to obtain corrected data to replace abnormal data on server. This control strategy is effective to reduce the amount of communication and improve the data quality.

#### 4.1.2. The Analysis of Data Consistency Checking

ARIMA is used to detect the anomaly in dissolved oxygen data set. The anomalous data in aquaculture can be classified into two types: One is the peak data which occurs occasionally. The other type is continuous data which deviates from the normal data for a period. In the experimental data set, there are 16 missing data caused by time delay or transmission error. Considering these two types of anomalous data, the confidence interval is set at 95%, and there are 16 anomalous data. Here, detection rate (*DR*) is used to evaluate the performance of anomaly detection.

(16) $$DR=\frac{TP}{TP+FN}\times 100\%,$$

where *TP* is the true positive number, and *FN* is the false negative number.

The *DR* of ARIMA is 93.75%. Then we need to utilize the data fusion mechanism to correct the anomalous data when failures occur.

### 4.2. Time Series Segmentation and Analysis

We separately evaluate the performance of fusion algorithm with two metrics, including Mean Absolute Error (MAE) and time [39]. MAE is computed by Equation (17).

(17) $$MAE=\frac{1}{N}\sum_{i=1}^{N}\left|y_{i}-{\hat{y}}_{i}\right|,$$

where *N* is the total number of sample points, *y_i_* is the real data and *ŷ_i_* is the fusion value. Then set the segment length in five days respectively. All experiments are implemented by MATLAB and run on a PC with 3.4 GHz Core (TM) processor, 4.0 G memory, and Microsoft Windows 7.

**Figure 4:**
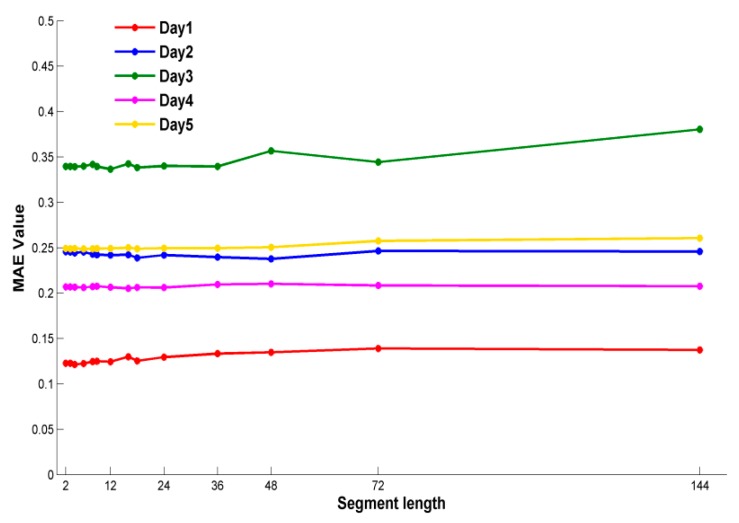
Mean Absolute Error (MAE) comparison of dynamic time warping time series strategy (DTWS)- improved support degree (ISD) at different segment length in five days.

**Figure 5:**
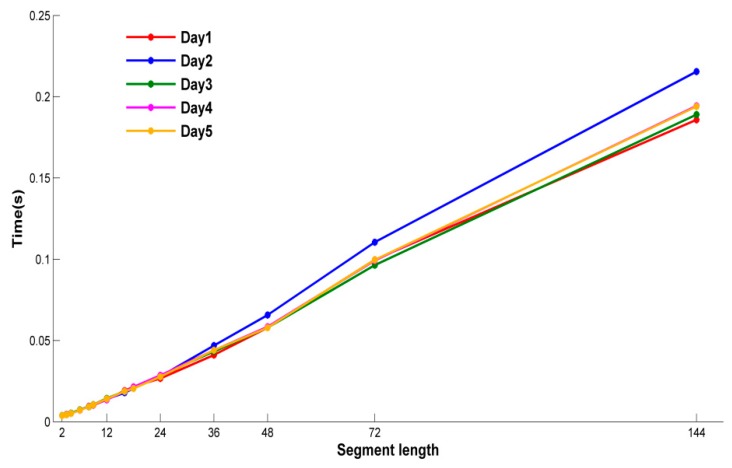
Time comparison of DTWS-ISD at different segment length in five days.

Figure 4 and Figure 5 show the MAE and time of different segment lengths in five days. As illustrated in Figure 4, the overall trend of MAE value is basically in a stable state with different segment lengths in five days. On the other hand, the run time is different obviously in Figure 5. The overall trend of run time varies with the segment length linearly. Considering these two metrics together, the segment length is 2, and the weighted fusion method with DTWS-ISD support function can obtain a stable MAE value in less time.

## 5. Results and Discussion

### 5.1. The Best Proposed Function

**Figure 6:**
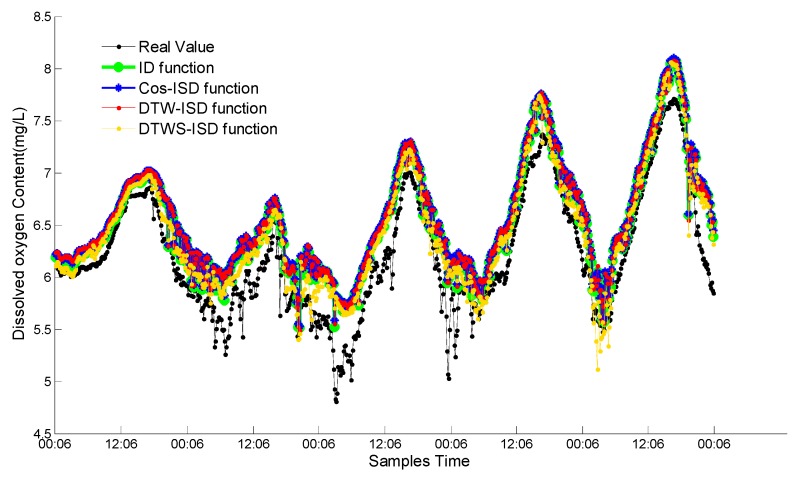
Weighted fusion results of four proposed functions.

To evaluate the DTW distance and time series segmentation strategy in DTWS-ISD method separately, we proposed other three functions: ISD, Cos-ISD (improved ISD by Cosine similarity [40,41]) and DTW-ISD. The cosine value of the angle is also introduced to replace the Euclidean distance and improve the ISD function. Figure 6 shows the fusion results of these functions. Here, *x* coordinate represents the different times (720 time points) in five days, *y* coordinate represents the dissolved oxygen content.

From Figure 6, the observed value of dissolved oxygen content basically has a periodical change trend every day, and some points deviate from the norm trend slightly during the sunrise. The changing trends of ID function, Cos-ISD function, DTW-ISD function and DTWS-ISD function are consistent with real values. However, the overall fusion result of DTWS-ISD function has the best approximation effect to the real values than the other three functions. The fusion results of the other three functions are close in Figure 6. In order to compare these functions sufficiently, we separately compare these functions with the metrics of MAE and time in Table 1.

From Table 1, we can see clearly that DTWS-ISD function has superior MAE value than other three functions and running in a short time. The relative MAE differences between DTWS-ISD and DTW-ISD, Cos-ISD, ISD are 4.8%, 53.6% and 23.1% in the test period respectively. The time of DTWS-ID is just 0.0039 s longer than ISD, but 2.4159 s shorter than DTW-ISD. That is because the proposed function can capture the continuity and fuzziness of data streams and improve the accuracy, but need take a little time.

The performance of Cos-ISD is unbalanced, regarding the maximal MAE value and shortest time. When compared with the other functions, it is not appropriate for Cos-ISD to finish fusion with the lowest accuracy. The results also show that DTW-ISD fusion precision is superior to ISD and Cos-ISD. DTW distance measuring algorithm can enhance the accuracy of ISD method greatly. However, the computing complexity of DTW distance is higher than cosine angle and Euclidean distance. DTWS-ISD has good performance both on accuracy and efficiency than DTW-ISD because of the time series segmentation strategy. It can reduce the complexity of DTW, thus improve the efficiency of DTWS-ISD. Considering both MAE and time, DTWS-ISD is the optimal fusion function.

### 5.2. Comparison with Existing Methods

**Figure 7:**
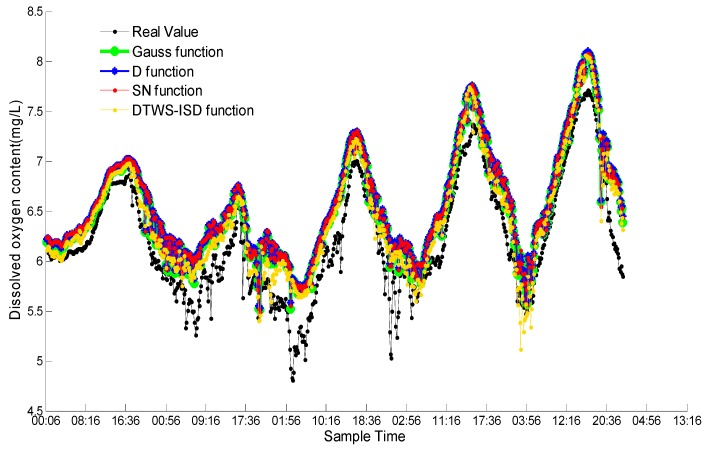
Comparison between DTWS-ISD with three existing functions.

In this experiment, we compare the performance of DTWS-ISD with Gauss support degree function [22], D function [23], and SN function [24]. Figure 7 shows the weighted fusion results of four functions. Here, *x* coordinate represents the different times in five days, and *y* coordinate represents the dissolved oxygen content.

From Figure 7, the changing curves of Gauss function, D function, SN function and DTWS-ISD function are consistent with real value. All curves have the periodic tendency of ascending first and descending in succession. However, the fusion results of DTWS-ISD are closer to the real value than other three existing functions. That is because DTWS-ISD can obtain better fusion results by exploring the correlation among sensors.

Meanwhile, the fusion results of these functions also have some data fluctuation during sunrise. The sunrise occurs at 5:00 a.m. to 7:00 a.m., and changes with the seasons. Although the results of weighting fusion with Gauss function, D function and SN function are close, there are still some gaps. The proximity of Gauss function to the real value is slightly better than D function and SN function. To verify the accuracy and efficiency of DTWS-ISD, we give the comparisons of MAE and run time of four functions in Table 2.

We can see clearly from Table 2, the MAE of DTWS-ISD is minimal, and the other three functions are closer to each other. The MAE value of DTWS-ISD is improved 24.07% of Gauss, 29.96% of D function and 29.58% of SN function. As for the run time, the gaps among these four functions were very narrow. The time of DTWS-ISD is 0.002 s longer than Gauss, 0.0026 s than D and 0.0031 s than SN. The results indicate that DTWS-ISD has a significantly more reliable performance and higher fusion precision than Gauss function, D function and SN function. It is obvious that the support degree function, optimized by DTW distance and time series segmentation strategy, is a good choice for improving the quality of dissolved oxygen data streams.

### 5.3. Analysis of Correlation between Sensors’ Distribution and Mutual Support Degree

**Figure 8:**
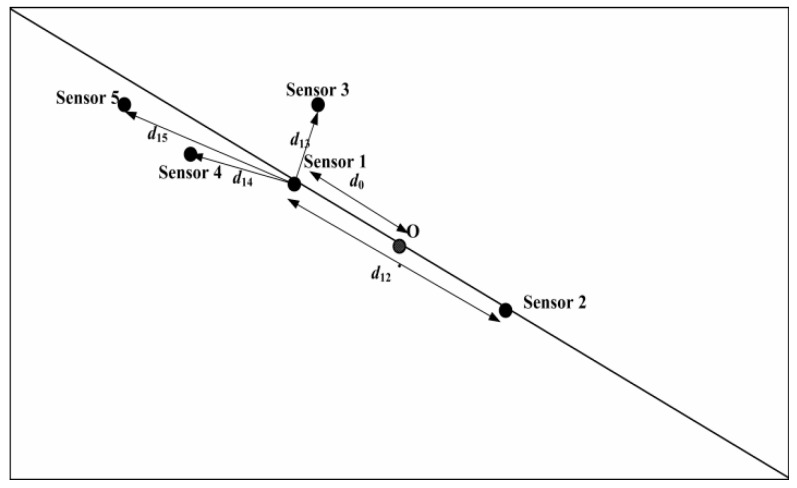
The distribution map of five sensors.

**Figure 9:**
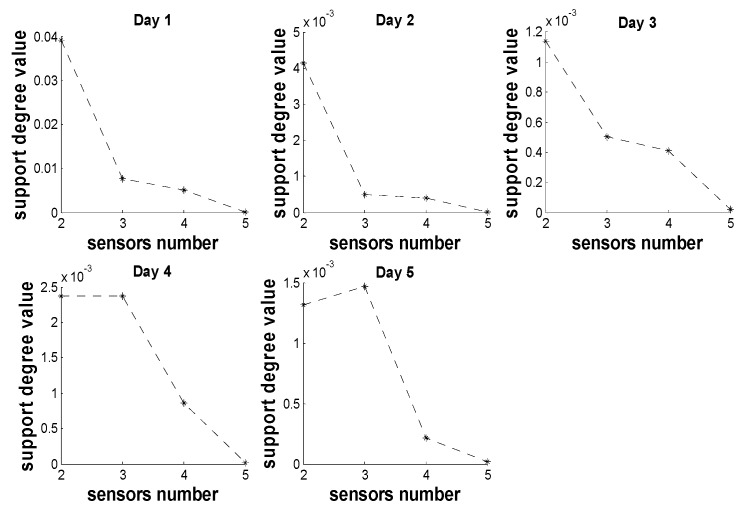
Support degree value of sensors to Sensor 1.

In the process of multi-sensors fusion, the locations of sensors influence the accuracy and reliability of data. Figure 8 shows the distribution map of five sensors in aquaculture pond. *D* = {*d*_12_, *d*_13_, *d*_14_, *d*_15_} (*d*_13_ < *d*_14_ < *d*_15_, *d*_12_ = 2*d*_0_) represents the distance between Sensor 1 and the other sensors, *d*_0_ denotes the distance between Sensor 1 and the center point *O*. Sensor 1 and Sensor 2 are distributed symmetrically to the point *O*. To analysis the correlation between sensor’s locations and mutual support degree, Figure 9 gives the support degree of four sensors to Sensor 1 over five days. Here, *x* coordinate denotes the sensors number, and *y* coordinate denotes the sensors’ support degree to Sensor 1.

Combined with the location feature in pond and distance *d*, the correlation is known by analyzing the Figure 8 and Figure 9. Sensor 2 and Sensor 1 are located from point *O* almost symmetrically, and the dissolved oxygen content distribution also have symmetrical feature around point *O* in Figure 8. From Figure 9, it is clear that Sensor 2 and Sensor 3 have greater support degree to Sensor 1. Meanwhile, the support degrees of Sensor 3, 4 and 5 are decreasing with the increase of distance *d_i_*.

However, the correlation between support degrees of Sensors and distance is not a linear relationship. It is also influenced by the location feature. The closer these sensors located to the shore of pond or corner, the more complex the impact is. In these positions, there are many microbes, aquatic plants and sludge, which result in the difference of the similarity between the data of Sensor 1 and the data of Sensor 3, 4, 5. Therefore, the support degree value largely depends on the distribution of sensors.

## 6. Conclusions

Multiple sensors deployed in different locations of aquaculture pond can provide complementary information for fault detection and correction. We provide a novel improved support degree function combining with weighted fusion method for enhancing data quality. This method comprises two techniques: One is the ISD function inspired by the theory of grey incidence analysis, which can reduce the computational complexity of Gauss support function. The other is DTW time series segmentation strategy that replaces Euclidean distance for both accuracy and efficiency. The experimental results demonstrate that DTWS-ISD function can realize the data fusion and correction efficiently. Performance analysis of DTWS-ISD shows that it performs better than other three counterparts (Gauss, D and SN support function) in term of MAE and time. Its effectiveness was verified in a real-world application for correcting the dissolved oxygen sensor data.

The following work will focus on two aspects. One is the improvement of DTWS-ISD function. It is expected to explore other algorithms to reduce computational complexity. The other is extending the idea of weighting fusion based on DTWS-ISD function to more fields, such as information prediction, target tracking, and data classification.

## Figures and Tables

**Table 1:** Comparison of four proposed functions.

Metrics	ISD	Cos-ISD	DTW-ISD	DTWS-ISD
Time(s)	0.0153	0.0063	2.4351	0.0192
MAE	0.3028	0.5018	0.2445	0.2328

**Table 2:** Performance comparison between DTWS-ISD and three existing functions.

Metrics	Gauss	D	SN	DTWS-ISD
Time(s)	0.0172	0.0166	0.0161	0.0192
MAE	0.3066	0.3324	0.3306	0.2328
